# Supplementary material for: Selenium intake in relation to all-cause and cardiovascular mortality in individuals with nonalcoholic fatty liver disease: A nationwide study in nutrition
Source: PLoS One. 2024 May 20;19(5):e0303140. doi: 10.1371/journal.pone.0303140 (PMC11104653; doi:10.1371/journal.pone.0303140)
Supplement: S1 Table — (DOCX) [file pone.0303140.s001.docx]

**S1 Table.** Least squares means of cardiac and liver metabolic markers according to selenium intake among participants with NAFLD

|  | **Selenium intake（μg/day）** | | | |  |
| --- | --- | --- | --- | --- | --- |
|  | **<78.0** | **78.0-111.5** | **111.6-150.9** | **>150.9** | ***P*_trend_ Power** |
| **CRP (mg/dL)** | 0.43±0.03 | 0.41±0.04 | 0.40±0.02 | 0.40±0.03 | 0.571 0.98 |
| **Uric acid (mg/dL)** | 5.49±0.09 | 5.45±0.07 | 5.44±0.10 | 5.47±0.09 | 0.879 0.86 |
| **Triglycerides (mg/dL)** | 139±13.3 | 162±12.9 | 188±13.6 | 165±12.8 | 0.217 0.92 |
| **HDL-C (mg/dL)** | 44.0±1.45 | 43.0±1.23 | 46.3±1.22 | 45.5±1.01 | 0.218 0.73 |
| **LDL-C (mg/dL)** | 43.4±3.66 | 48.8±4.98 | 44.8±5.46 | 58.9±5.03 | 0.007 0.81 |
| **Glucose (mmol/L)** | 5.47±0.11 | 5.71±0.16 | 5.76±0.16 | 5.74±0.13 | 0.172 0.67 |
| **HbA1c (%)** | 5.38±0.09 | 5.50±0.09 | 5.62±0.08 | 5.53±0.0 | 0.253 0.88 |
| **HOMA-IR** | 3.45±0.42 | 3.75±0.50 | 4.12±0.35 | 4.91±0.65 | 0.022 0.91 |
| **AST (U/L)** | 25.2±1.43 | 24.7±1.03 | 24.7±0.90 | 24.3±1.25 | 0.496 0.96 |
| **ALT (U/L)** | 23.9±1.57 | 23.9±1.54 | 24.2±1.24 | 25.0±1.85 | 0.588 0.92 |

CRP, C-reactive protein; HDL-C, high-density lipoprotein cholesterol; LDL-C, low-density lipoprotein cholesterol; HbA1c, glycated hemoglobin A1c; HOMA-IR: HOMA of insulin resistance; AST, aspartate aminotransferase; ALT, alanine aminotransferase.

Least-square (mean ± standard error) was estimated using general linear model with adjustment of age (continuous), sex (male or female), race (non-Hispanic White, non-Hispanic Black, Mexican American, or other), education (less than high school, high school or equivalent, or college or above), physical activity (inactive, insufficiently active, or active), smoking status (never, former, or current smoker), body mass index (kg/m^2^; <25.0, 25.0-29.9, or ≥30.0), family income-poverty ratio (≤1.30, 1.31-3.50, or >3.50), Healthy Eating Index (continuous). Power is calculated using the R language “pwr” package.
