# Supplementary material for: Selenium intake in relation to all-cause and cardiovascular mortality in individuals with nonalcoholic fatty liver disease: A nationwide study in nutrition
Source: PLoS One. 2024 May 20;19(5):e0303140. doi: 10.1371/journal.pone.0303140 (PMC11104653; doi:10.1371/journal.pone.0303140)
Supplement: S2 Table — (DOCX) [file pone.0303140.s002.docx]

**S2 Table.** HRs (95% CIs) of mortality according to selenium intake after excluding participants with NAFLD who had stroke or heart failure at baseline in NHANES III

|  | **Selenium intake（μg/day）** | | | |  | **One-unit increment in**  **log-transformed selenium intake** |
| --- | --- | --- | --- | --- | --- | --- |
|  | **<78.0** | **78.0-111.6** | **111.7-152.1** | **>152.1 *P*_trend_** | |  |
| **All-cause mortality** | | |  |  |  |  |
| No. deaths/total | 174/631 | 127/583 | 127/489 | 113/511 |  |  |
| **Model 1**  HR (95% CI) | 1 | 0.71 (0.5-0.99) | 0.73 (0.48-1.1) | 0.65 (0.47-0.88) | 0.016 | 0.71 (0.45-1.11) |
| *P*-value |  | 0.046 | 0.137 | 0.006 |  | 0.135 |
| **Model 2**  HR (95% CI) | 1 | 0.61 (0.41-0.9) | 0.67 (0.41-1.09) | 0.65 (0.47-0.92) | 0.061 | 0.71 (0.43-1.16) |
| *P*-value |  | 0.012 | 0.104 | 0.015 |  | 0.173 |
| **Cardiovascular mortality** | | |  |  |  |  |
| No. deaths | 52 | 25 | 27 | 34 |  |  |
| **Model 1**  HR (95% CI) | 1 | 0.25 (0.13-0.48) | 0.59 (0.27-1.31) | 0.45 (0.21-0.99) | 0.155 | 0.56 (0.21-1.47) |
| *P*-value |  | <0.001 | 0.196 | 0.047 |  | 0.237 |
| **Model 2**  HR (95% CI) | 1 | 0.18 (0.08-0.44) | 0.37 (0.17-0.81) | 0.39 (0.18-0.85) | 0.119 | 0.35 (0.12-1.03) |
| *P*-value |  | <0.001 | 0.013 | 0.018 |  | 0.056 |

NAFLD, nonalcoholic fatty liver disease; NHANES III, the Third National Health and Nutrition Examination Survey; CIs, confidence intervals; HRs, hazard ratios. Cox proportional hazards models were used to estimate the HRs (95% CIs) for mortality according to selenium intake.

Model 1 Adjusted for age (years), sex (male or female), and self-reported race (non-Hispanic White, non-Hispanic Black, Mexican American, or others).

Model 2 Further adjusted for education (less than high school, high school or equivalent, or college or above), Healthy Eating Index (continuous), family income-poverty ratio (≤1.30, 1.31-3.50, or >3.50), physical activity (inactive, insufficiently active, or active), smoking status (never, former, or current smoker), body mass index (kg/m^2^; <25.0, 25.0-29.9, or ≥30.0), dyslipidemia (yes or no), diabetes (yes or no), hypertension (yes or no), Fibrosis-4 index (<1.30, 1.30-2.66, ≥2.67).
