# Supplementary material for: Selenium intake in relation to all-cause and cardiovascular mortality in individuals with nonalcoholic fatty liver disease: A nationwide study in nutrition
Source: PLoS One. 2024 May 20;19(5):e0303140. doi: 10.1371/journal.pone.0303140 (PMC11104653; doi:10.1371/journal.pone.0303140)
Supplement: S3 Table — (DOCX) [file pone.0303140.s003.docx]

**S3 Table.** HRs (95% CIs) of mortality according to selenium intake after excluding participants with NAFLD who died within the first five years of follow-up in NHANES III

|  | **Selenium intake（μg/day）** | | | |  | **One-unit increment in**  **log-transformed selenium intake** |
| --- | --- | --- | --- | --- | --- | --- |
|  | **<78.1** | **78.1-111.7** | **111.8-152.0** | **>152.0 *P*_trend_** | |  |
| **All-cause mortality** | | |  |  |  |  |
| No. deaths/total | 175/645 | 133/595 | 124/490 | 112/511 |  |  |
| **Model 1**  HR (95% CI) | 1 | 0.76 (0.52-1.1) | 0.79 (0.54-1.16) | 0.75 (0.54-1.03) | 0.136 | 0.75 (0.48-1.2) |
| *P*-value |  | 0.142 | 0.233 | 0.077 |  | 0.231 |
| **Model 2**  HR (95% CI) | 1 | 0.66 (0.44-0.99) | 0.73 (0.45-1.16) | 0.78 (0.54-1.12) | 0.351 | 0.78 (0.47-1.32) |
| *P*-value |  | 0.042 | 0.182 | 0.171 |  | 0.361 |
| **Cardiovascular mortality** | | |  |  |  |  |
| No. deaths | 52 | 29 | 25 | 34 |  |  |
| **Model 1**  HR (95% CI) | 1 | 0.3 (0.19-0.48) | 0.73 (0.34-1.56) | 0.7 (0.33-1.48) | 0.708 | 0.71 (0.23-2.18) |
| *P*-value |  | <0.001 | 0.416 | 0.347 |  | 0.548 |
| **Model 2**  HR (95% CI) | 1 | 0.23 (0.12-0.43) | 0.47 (0.22-1) | 0.56 (0.26-1.21) | 0.533 | 0.44 (0.12-1.6) |
| *P*-value |  | <0.001 | 0.051 | 0.141 |  | 0.212 |

NAFLD, nonalcoholic fatty liver disease; NHANES III, the Third National Health and Nutrition Examination Survey; CIs, confidence intervals; HRs, hazard ratios. Cox proportional hazards models were used to estimate the HRs (95% CIs) for mortality according to selenium intake.

Model 1 Adjusted for age (years), sex (male or female), and self-reported race (non-Hispanic White, non-Hispanic Black, Mexican American, or others).

Model 2 Further adjusted for education (less than high school, high school or equivalent, or college or above), Healthy Eating Index (continuous), family income-poverty ratio (≤1.30, 1.31-3.50, or >3.50), physical activity (inactive, insufficiently active, or active), smoking status (never, former, or current smoker), body mass index (kg/m^2^; <25.0, 25.0-29.9, or ≥30.0), dyslipidemia (yes or no), diabetes (yes or no), hypertension (yes or no), Fibrosis-4 index (<1.30, 1.30-2.66, ≥2.67).
